# Three‐dimensional morphological revealing of human placental villi with common obstetric complications via optical coherence tomography

**DOI:** 10.1002/btm2.10372

**Published:** 2022-07-27

**Authors:** Guangming Ni, Junming Zhong, Xuemei Gao, Renxiong Wu, Wenjing Wang, Xiaoshan Wang, Yao Xie, Yong Liu, Jie Mei

**Affiliations:** ^1^ School of Optoelectronic Science and Engineering University of Electronic Science and Technology of China Chengdu China; ^2^ Department of Obstetrics and Gynaecology Sichuan Provincial People's Hospital, University of Electronic Science and Technology of China Chengdu Sichuan China; ^3^ Cancer Center of University of Electronic Science and Technology of China and Sichuan Provincial People's Hospital Chengdu China

**Keywords:** common obstetric complications, optical coherence tomography, placental villi, three‐dimensional morphology

## Abstract

Placental villi play a vital role in human fetal development, acting as the bridge of material exchange between the maternal and fetal. The abnormal morphology of placental villi is closely related to placental circulation disorder and pregnancy complications. Revealing placental villi three‐dimensional (3D) morphology of common obstetric complications and healthy pregnancies provides a new perspective for studying the role of the placenta and its villi in the development of pregnancy diseases. In this study, we established a noninvasive, high‐resolution 3D imaging platform via optical coherence tomography to reveal placental villi 3D morphological information of diseased and normal placentae. For the first time, 3D morphologies of placental villous tree structures in common obstetric complications were quantitatively revealed and corresponding 3D information could visualize the morphological characteristics of the placental villous tree from a more intuitive perspective, providing helpful information to the study of fetal development, feto‐maternal material exchange, and gestational complications treatment.

## 1 INTRODUCTION

Placenta is a feto‐maternal organ that provides oxygen and nutrients to the fetus and removes waste products from the fetus' blood.^1^, ^2^ The key part of the placenta is the villous tree which sprouts from the chorionic plate into the intervillous space.^3^ The branches of the stems continue branching, leading to a large number of stem villi generations and further branches, finally ending as freely floating villi in the intervillous space. Stem villi^4^ have the largest diameter, which serves as the mechanical support for the villi tree and plays a small role in the exchange of fetal material. From the intermediate villi branching from the stem villi sprout terminal villi, which are the most important components of the villous tree.^5^ Villous trees, acting as part of the border between maternal and fetal blood during pregnancy, increase the area of the chorionic membrane across where oxygen, carbon dioxide, and other substances can diffuse between the maternal and fetal blood.^2^

Many pregnancy complications are related to the maldevelopment of the placental villous tree.^6^ Material exchanging between maternal and fetus in the placenta cannot provide the fetus with the nutrients needed for normal growth, which is considered to be a possible cause of fetal growth restriction (FGR).^7^ Gestational hypertension (GH), one category of hypertensive disorders of pregnancy (HDP) that is one of the leading causes of morbidity and mortality, can be dangerous for both the mother and fetus. Although the cause of GH is remaining unclear now, it has been reported that the placenta villi with GH have abnormalities in histological features.^8^ Gestational diabetes mellitus (GDM) is a type of diabetes that develops during pregnancy and causes many structural and functional changes in the placenta villi.^9^ Therefore, revealing the three‐dimensional (3D) morphology of multi‐diseased placental villi is of great significance for understanding the interaction and influence of placental villi with gestational complications, as well as for studying the fetal development, feto‐maternal material exchange, and gestational complications treatment.

Up to now, three‐dimensional (3D) visualizing human placental villous tree structures are limited. Most of our understanding of placental villi is based on histologic analysis^10^ of stained thin sections from delivered placenta specimens. However, histological section as a two‐dimensional (2D) method, cannot present the 3D morphology of placental villi, and also time‐costing. Scanning electron microscopy (SEM)^11^ is used to view placenta microstructure subjects to artifacts and has the problems of narrow field of view and limited depth of focus. Confocal laser scanning microscopy (CLSM)^12^ which needs to perfuse and stain the sections, like the histological section, is also faced the same limitations. Synchrotron X‐ray imaging^13^ is used to generate high‐resolution massively multiscale datasets of the human placenta. However, it requires complex fixation, perfusion, staining, and embedding operation before imaging. Micro‐CT^14^ can provide volume imaging with micrometer scale resolution but magnification is at the cost of a limited field of view. The lack of 3D morphological revealing of placenta villi limits our ability to view the villous tree as a whole and characterize many morphological features of the villous tree, such as branches and villi morphology.

Here we proposed a noninvasive, high‐resolution optical coherence tomography (OCT)^15^ imaging platform to reveal 3D morphological information on the villous tree structure of healthy human placentae and human placentae with common obstetric complications. As an emerging technology for biomedical imaging technology, OCT employing near‐infrared light low‐coherence interferometry, can achieve non‐destructive, micron‐level resolution 3D imaging of bio‐tissue,^16^, ^17^ and has been widely used for biomedical imaging and clinical diagnosis, such as imaging human retinal 3D microstructures in vivo. In this study, we employed a noninvasive 3D imaging platform via OCT to reveal the 3D morphology of multi‐diseased placental villi and extracted high‐resolution structural and morphological information from three‐category gestational complications and normal placentae for the first time. In particular, we quantitatively extracted the morphological characteristics of the placental villous tree structure including branches and villi morphology for each diseased and normal placenta which are significant to study the effect of pregnancy complications on the morphology of the placental villi. Moreover, we also first revealed the villous tree morphology of the two‐gestational‐comorbidities placenta, providing a new perspective for studying the relationship and mutual influence of gestational complications. Our results quantitatively revealed the 3D morphology of placental villi with different gestational complications and healthy pregnancies and provided additional key information for study on the influence of placental villi morphology on gestational complications and fetal development.

## 2 MATERIALS AND METHODS

### 2.1 Sample preparation

This research was approved by the Ethics Committee of the University of Electronic Science and Technology of China (ID: 1061420211102003), and the placenta sample acquisition and preparation were conducted in the Department of Obstetrics and Gynecology, Sichuan Provincial People's Hospital, the affiliated hospital of the University of Electronic Science and Technology of China, with written informed consent from pregnant women. All pregnant women met the following criteria: (1) Singleton pregnancies, aged 23–35 years; (2) No fetal malformations, chromosomal abnormalities, congenital infection; (3) No pre‐pregnancy medical basic disease, no history of smoking, alcohol, substance abuse, or psychiatric disorders; (4) Regular birth examination during pregnancy; and (5) definite diagnosis of the corresponding disease in the obstetric outpatient clinic. We collected placentae from six cases of the healthy pregnancy, six cases of the pregnancy with HDP, eight cases of the pregnancy with GDM, and six cases of the pregnancy with FGR including two cases of the pregnancy complicated with FGR and GDM.

**FIGURE 1:**
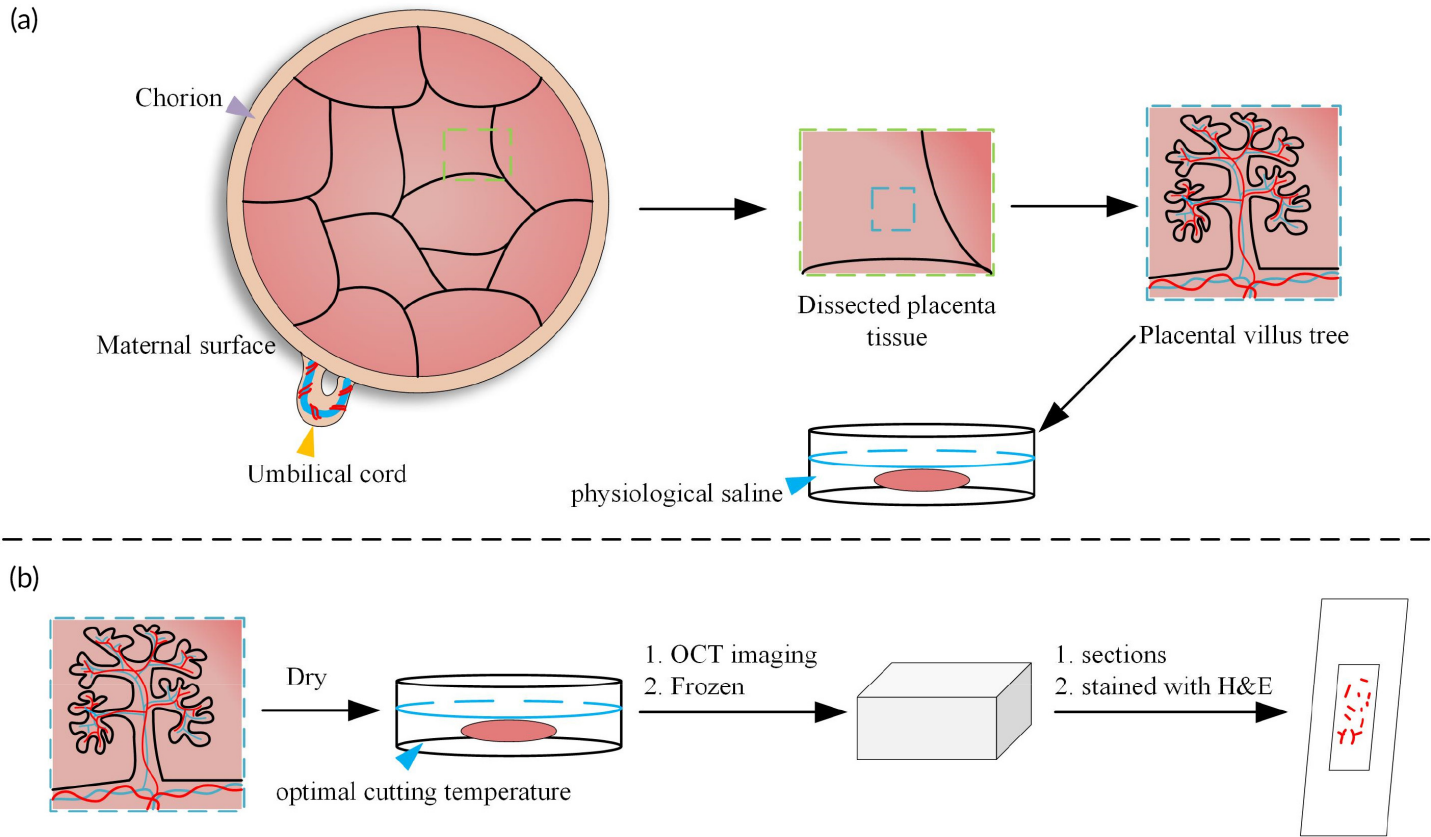
Villous tree acquirement after delivery. (a) Schematic diagram of placenta villus tree acquiring. Several placental villous trees (blue dashed box) were dissected from placenta tissue (green dashed box). Placental villous tree sprouts from chorion (violet arrow) connected with fetal by the umbilical cord (yellow arrow). (b) Schematic diagram of histological sections. Placental villous trees were dried and then embedded in optimal cutting temperature medium to OCT imaging. Then frozen immediately to conduct histological sections. OCT, optical coherence tomography

The placental tissues were randomly obtained from the maternal surface of the placenta after the placenta delivery in 1 h, as Figure 1 shows. The placental tissues in each case were immersed in 0.9% physiological saline to maintain morphology and immediately transported to the laboratory with ice bags using a biological sample delivery box. Dissected villus tree indicated by the blue dashed box in Figure 1a isolated from delivered placental tissue indicated by the green dashed box in Figure 1a after fetal blood was cleared from the tissue with physiological saline. At least four placental tissues were obtained from each placenta, and more than 20 dissected villus tree regions were isolated from each placenta tissue for 3D imaging. More than 80 villus tree regions were obtained from each placenta sample to ensure the results of 3D imaging of placental villous trees are more general.

To compare the imaging performance of OCT and histological sections, we prepared a healthy placenta sample to implement both OCT and histological sections, as Figure 1b shows. We collected and acquired the placenta sample following the same criteria and transported it to the laboratory. First, we performed OCT imaging of the placenta villi. Placental villous tissue was removed from the 0.9% normal saline, dried on the absorbent paper towel, and then embedded in an optimal cutting temperature medium to acquire OCT images. After OCT imaging, optical cutting temperature embedded placental villous was quickly placed on a freezing microtome for snap freezing. We used Leica Biosystems cryostat to prepare sections and optimal cutting temperature embedded villous were serially frozen‐sectioned at 10 μm thick along the longitudinal direction. The sections were first stained with hematoxylin for 10 s and washed with dripping water, then stained with eosin for 150 s and washed again with dripping water for 1–2 s. Different concentration gradients of alcohol were used for dehydration. Next, we used xylene for transparent treatment. Finally, the sheets were blocked with neutral gum. The stained sections were observed under a microscope, and two‐dimensional images of a suitable region were acquired.

### 2.2 3D imaging platform

**FIGURE 2:**
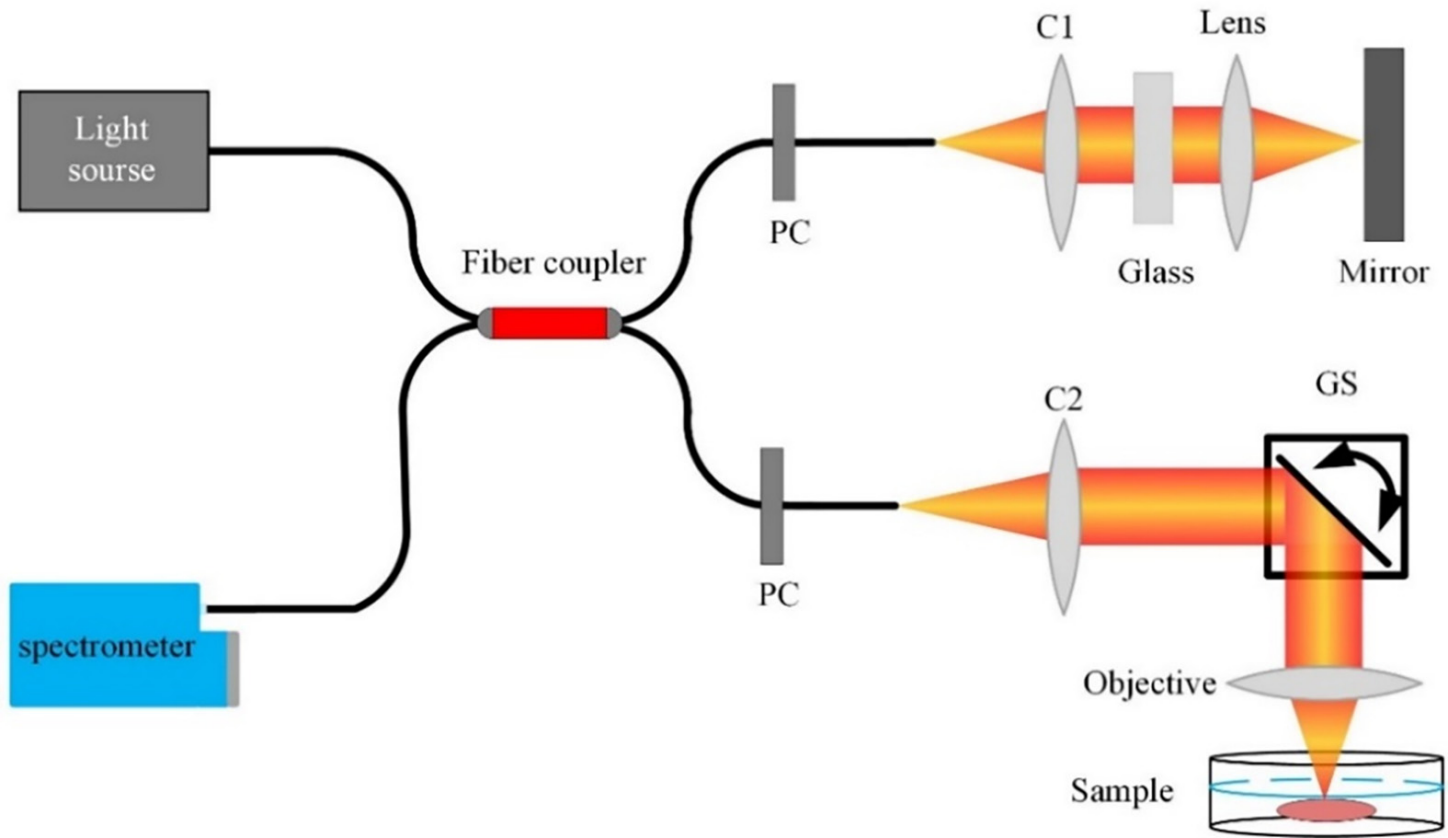
OCT system used for revealing placental villi 3D morphology. C1–C2, collimator; GS, galvanometer scanner; PC, polarization controller; OCT, optical coherence tomography

Here a spectral‐domain OCT system was used as the 3D imaging platform for the placenta villi's three‐dimensional morphological revealing, as Figure 2 shows. The spectral‐domain OCT setup used a superluminescent diode‐based broadband light source (cBLMD‐T‐850‐HP, Superlum) with a center wavelength of 855 nm and a 160 nm bandwidth, a 2048‐pixel spectrometer (Cobra‐S 800, Wasatch Photonics), a 2‐axis scanning galvanometer scanner (GVSM002‐EC/M, Thorlabs), a 2 × 2 850‐nm wideband fiber optic coupler (TW850R5A2, Thorlabs), two identical collimators (TC25APC‐850, Thorlabs), and two polarization controllers (FPC030, Thorlabs), one for the sample arm and one for the reference arm. 4× objective (Olympus) lens was configured when imaging samples. The axial and transverse optical resolutions were ~2.3 μm in air and ~6.9 μm, respectively. The OCT imaging exposure time 𝑡_𝑒_ used was 25.0 μs, and both the A‐scan number per B‐scan and B‐scan number used were 800. The light power on the sample was ~8.9 mW and the sensitivity of the OCT system was ~102 dB. The maximum imaging depth into the villi was ~1.7 mm.

### 2.3 Data processing

OCT images of the villous tree of placenta with three different gestational complications and healthy pregnancy were acquired immediately after delivery and transported from the hospital to the lab room. OCT scanning range was adjusted to acquire a broad view of villous trees. The actual scanning range was ~4.08×4.08 mm^2^, and the corresponding transverse pixel resolutions was ~5.1 μm, respectively. Obtained OCT datasets of villous trees were first processed to generate OCT structural images with rescaling operation in the axial direction to achieve the same pixel resolution as that in the transverse direction. Each 3D morphology image of placenta villi was acquired via the OCT imaging platform and further processed via ImageJ^18^ (Version 1.53c, US National Institutes of Health). Here the rescale operation of OCT images was implemented by using the ImageJ scale operation. Here we used a 0.3333 index to rescale the depth axis to achieve the same resolution with the transverse plane to acquire an accurate 3D effect of placenta villi. We used the ‘scale’ method of ImageJ and adjust the Y scale to 0.3333.

### 2.4 Quantification of villi parameters

**FIGURE 3:**
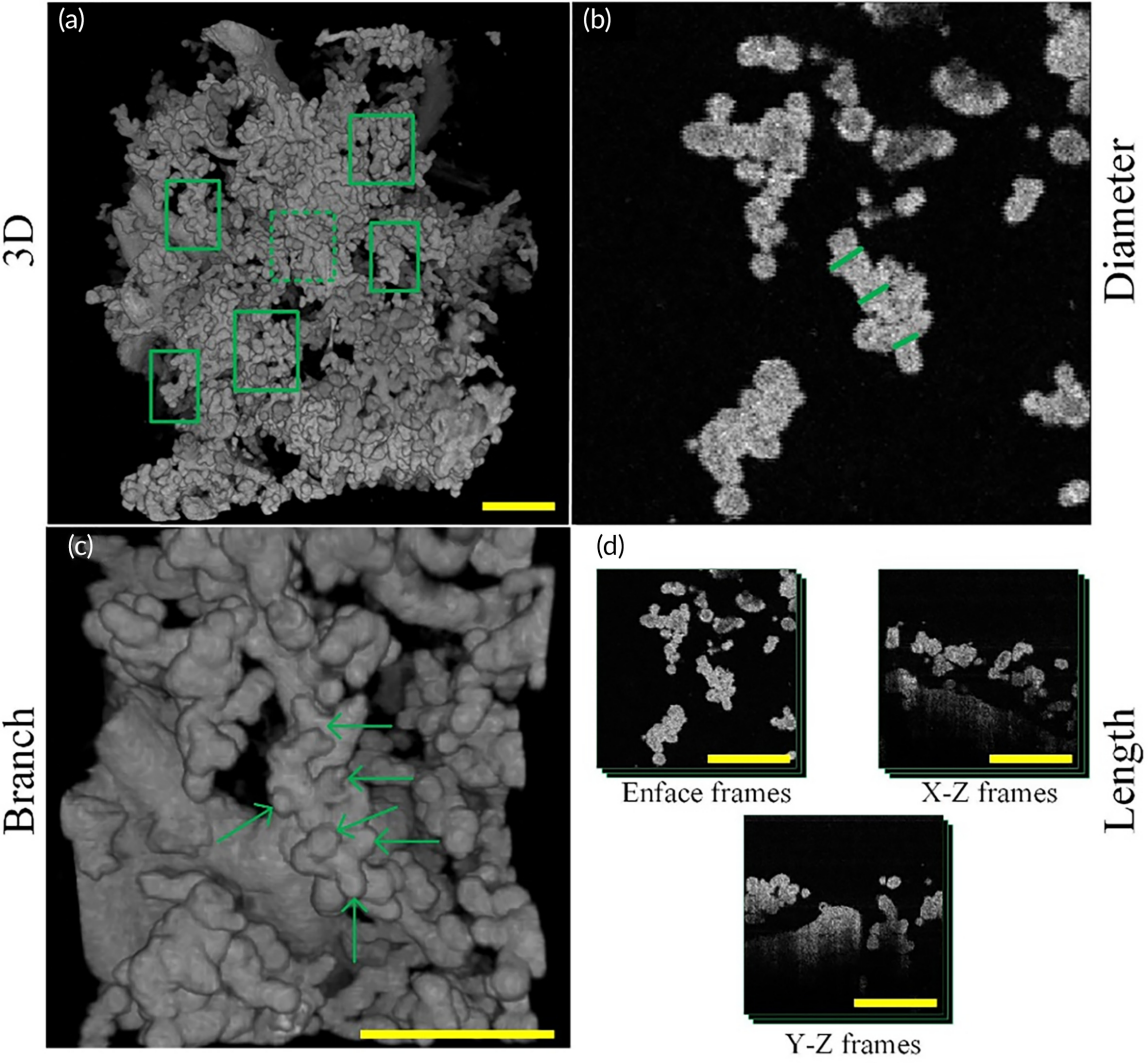
Schematic diagram of placenta villi quantification. (a) Schematic diagram of placenta villi sampling locations. Six villi were selected to quantify villi morphological parameters indicated by green boxes in a 3D OCT image of placental villi. (b) Diameter quantification of placenta villi. Green lines in OCT enface image corresponding to the region of green dashed box in (a) indicate villi diameter quantification sampling location. Three locations of every intermediate villus were selected. (c) Villi branches quantification of placenta villi. 3D OCT image of placenta villi corresponding to the region indicated by the green dashed box in (a). Six green arrows indicate six branches of the intermediate villi in this view. We also perform the rotation in 3D to count villi branches more accurately. (d) Length quantification of placenta villi. Frame numbers where quantified villi appeared were counted in three different cross‐sections. OCT, optical coherence tomography

Here we quantitatively evaluated the 3D tree structure morphological parameters of placental villi with or without pregnancy complications, as follows: the diameter of intermediate villi and terminal villi, the length of terminal villi, and the number of villi branches. Here we quantitatively evaluated the morphological parameters of healthy and complicated placenta villi, and for each condition, we quantified four different placenta samples from four different pregnancy mothers. For each sample, we quantitatively analyzed the morphological parameters of six placenta villus tree regions which were evenly selected from one OCT 3D dataset, as Figure 3a shows.

To calculate the diameters of terminal and intermediate villi of the placental villous tree, five different cross‐sectional images of corresponding villi were selected. The diameter of placental villi was calculated in five frames and performed the average to acquire the result. Three different quantified locations of villi diameter were shown (Figure 3b).

To quantify the number of villi branches, we used 3D OCT images of corresponding villi and villi branches that could easily be observed in 3D images, as Figure 3c shows. For each villus, we counted the branches of it three times and take the average to get the result. We also performed the rotation to count the branch number accurately. To quantify the length of placental villi, we calculated three cross‐sectional images in the XY, XZ, and YZ planes, selecting all frames where the selected placental villi appeared, as Figure 3d shows. The length of terminal villi L is approximately calculated as:

$$L\approx {\sqrt{F_{\mathrm{XY}}^{2}+F_{\mathrm{XZ}}^{2}+F_{\mathrm{YZ}}^{2}}}^{*}\delta$$

where $F_{\mathrm{XY}}$, $F_{\mathrm{XZ}}$, $F_{\mathrm{YZ}}$ was frame number where the selected placental villi were present in XY, XZ, YZ cross‐sectional images, respectively, δ was the rescaled resolution of OCT images.

## 3 RESULTS

Here we acquired images of human placental villi with a high‐resolution 3D imaging platform via OCT. For the first time, 3D villous tree structures of placentae with GH, GDM, FGR, multiple comorbidities, and healthy pregnancies were revealed. Placental villi morphology with pregnancy diseases is different from normal placental villi, showing that gestational complications have different effects on villi morphology. To reveal the 3D morphologies of placentae with different complications, for each sample, we imaged more than 80 placental villus tree regions via the 3D imaging platform. We revealed placental villus tree 3D morphology via OCT and showed morphology by providing a top view image of the OCT 3D image, one supplementary video of OCT 3D morphology, and three different OCT sections images. Specifically, we revealed two different orientations OCT B‐scan images which is the two‐dimensional OCT cross‐sectional image of the sample acquired by performing two‐dimensional scanning at several positions, and one representative *en*‐face section image which is a transverse two‐dimensional cross‐sectional image at a fixed axial depth position. To compare the imaging performance of OCT and histological sections, we also selected one healthy pregnancies placenta sample to conduct both OCT imaging and histological sections are shown below.

### 3.1 OCT versus histological sections

**FIGURE 4:**
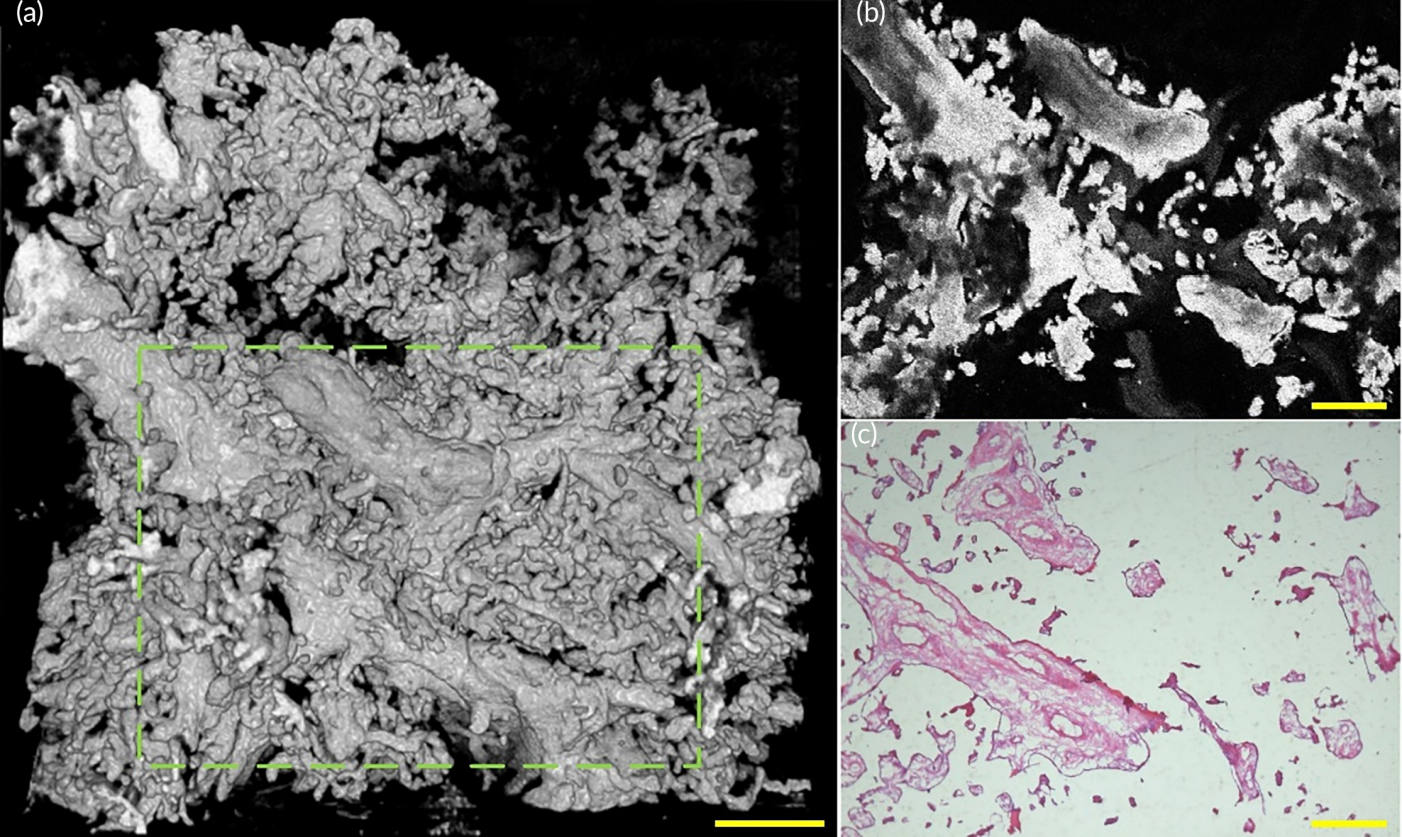
OCT and histological sections images of placenta villi of placenta with healthy pregnancy. (a) Top view of 3D OCT image of a healthy placenta sample. (b) A representative OCT *en*‐face image of the healthy placental sample showed the *en*‐face image corresponding to the area indicated by the green dashed box in (a). (c) A histological section image of the healthy placental sample corresponding to the area indicated by the green dashed box in (a). OCT, optical coherence tomography

Here we implemented histology sections on a placenta sample of healthy pregnancy to compare the imaging performance between histological sections and OCT, as Figure 4 shows. A top view of a 3D OCT image of a healthy placental villous tree revealed placental villi 3D morphology, as Figure 4a shows. The representative magnified OCT *en*‐face image of placenta villi of healthy pregnancies is shown in Figure 4b, which corresponds to the region indicated with the green dashed boxes in Figure 4a. In the OCT *en*‐face image of the placenta villous tree region, the villi distribution of placenta villous tree structures was observed clearly. The placenta villus tree structure of the healthy placenta of OCT *en*‐face images are identified in hematoxylin and eosin (H&E) stained tissue slide images (Figure 4c) that show 2D morphological features of the placental villi.

The high resolution of OCT *en*‐face images makes it possible to image placental villi microstructure at a scale that is comparable to histopathology, without the complex sample preparation. The comparison between OCT *en*‐face images and histological sections images of healthy placental villus trees demonstrated the advantage of OCT to allow 3D visualization of the placental villous tree. Furthermore, as shown in Figure 4a, OCT can not only provide depth‐resolved 2D *en*‐face images which are comparable to histopathology but also can reveal high‐resolution 3D morphology images simultaneously. OCT could provide non‐invasive, high‐resolution 3D images of placental villi, having simplifier sample processing compared to time‐costing histological sections which need to perfuse and stain the sections before imaging.

### 3.2 Healthy pregnancy

**FIGURE 5:**
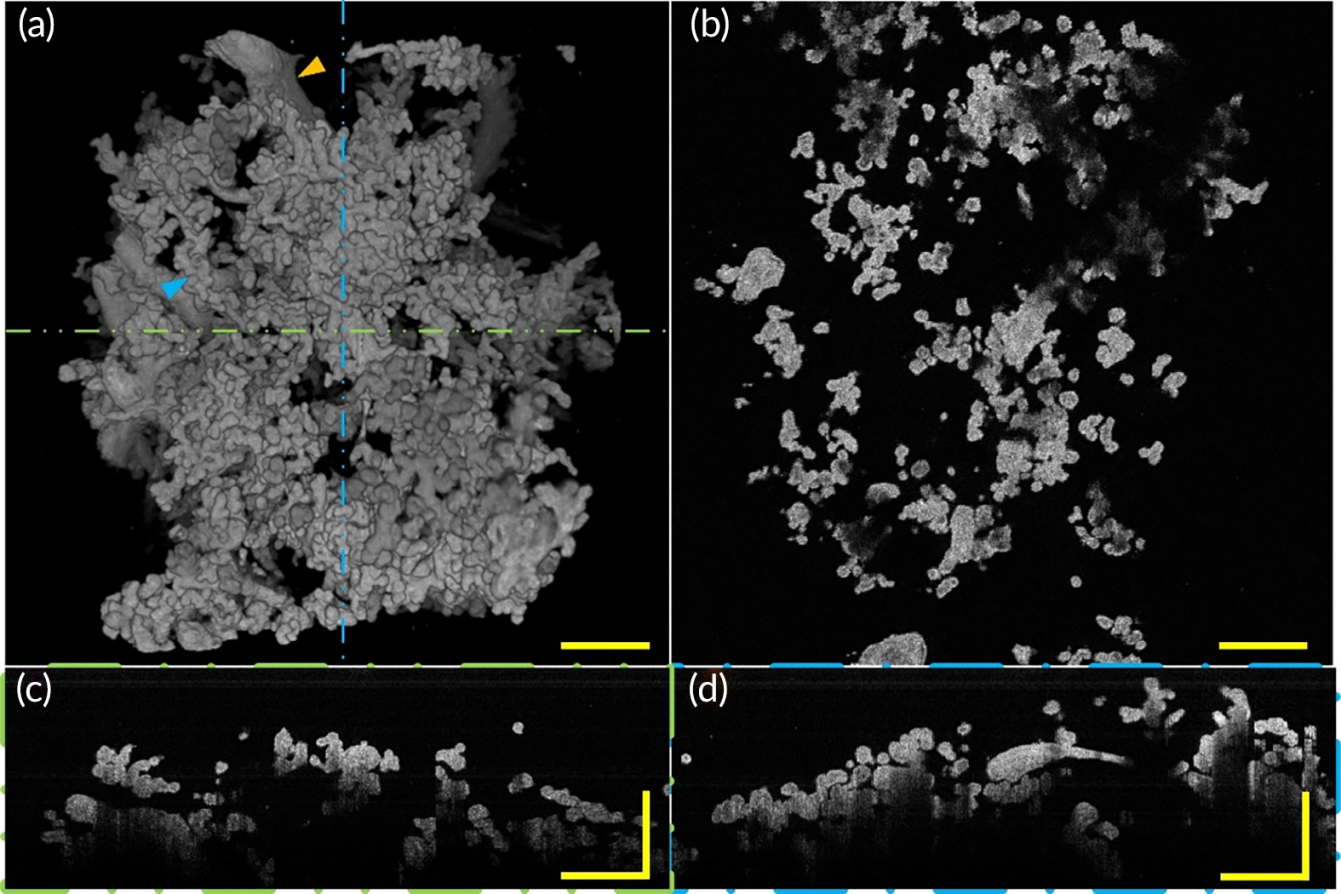
3D morphology and section images of placenta villi of placenta with healthy pregnancy via OCT. (a) Top view of 3D OCT morphology shows healthy placenta has villi varied in diameter and the tree structure of the uncomplicated placenta is well‐developed, with mature intermediate villi and well‐branched terminal villi. Three different types of villi morphology can be observed: larger diameter stem villi that serve to support the structure of the villi tree (orange arrow) and a slightly smaller diameter intermediate villi with densely branched grape‐like terminal villi on its surface (blue arrow). (b) A representative OCT en‐face image of healthy placental villi. (c) A representative rescaled B‐scan image of placental villi corresponding to the position indicated by the green line in (a). (d) A representative rescaled B‐scan image of placental villi corresponding to the position indicated by the blue dashed line in (a). All scale bars are 500 μm. OCT, optical coherence tomography

Histology method indicates that placental villi from full‐term women without comorbidities are densely branched, with tightly packed adjacent villous shafts, and numerous villi.^4^ Figure 5a–d and Video S1 reveal the morphology of a placenta of healthy pregnancy. A top view of a 3D OCT image of the normal placental villous tree revealed villi morphology of healthy pregnancy placenta sample, as Figure 5a shows.

Here we revealed three‐types villi of the villous trees. Stem villi serve as the mechanical support for the villi tree but play a small role in material exchange.^4^ Grape‐like terminal villi densely branched from intermediate villi serve as the functional unit of the placenta, transfer materials between mother and fetus. In this paper, we focused on the relationship between villi morphology and gestational complications, especially the possible influence of placental villi morphology on the exchange of materials between mother and infant. Therefore, here we paid more attention to intermediate villi and terminal villi. We observed placental villous trees of healthy pregnancies are densely branched with well‐developed intermediate villi from which numerous terminal villi sprouted. A representative OCT en‐face image of healthy placental villi is shown in Figure 5b. In longitudinal‐plane B‐scan images (Figure 5c,d) corresponding to the positions represented by the green and blue dashed lines in Figure 5a, the villus diameters of healthy pregnancies could be revealed. Combining the three‐dimensional morphology, we can find that there were intermediate villi and terminal villi. The villi with relatively large diameters are intermediate villi and small diameters villi are terminal villi. In these images, we observed villi characteristic that is consistent with the results of histological sections. Three‐dimensional volume rendering of the OCT data showed villous tree microstructure that is similar to what one might see by SEM. Furthermore, more villous tree branching and morphology information can be acquired via our OCT imaging platform because we have a wider field of view compared with SEM.

### 3.3 Gestational hypertension

Gestational hypertension (GH) is one of the most common categories of HDP. HDP is a complex diseases category that can be differentiated into many disorders for each will cause different gestational outcomes.^19^ GH is the development of newly onset hypertension after 20 weeks of gestation in the absence of diagnostic criteria for preeclampsia and has a risk of progression to preeclampsia. The etiology of HDP remains controversial but now accepted that it is associated with the maldevelopment of the placental villous and their blood vessels.^20^, ^21^ Because of placental is in an anoxic environment, it results in poor villous development. Histological sections study indicated placenta with HDP has a low number of villi exhibiting long, thin, or filamentous terminal villi with minimal branching, and often accompanied by a lack of terminal villi or a very small villus diameter.^22^

**FIGURE 6:**
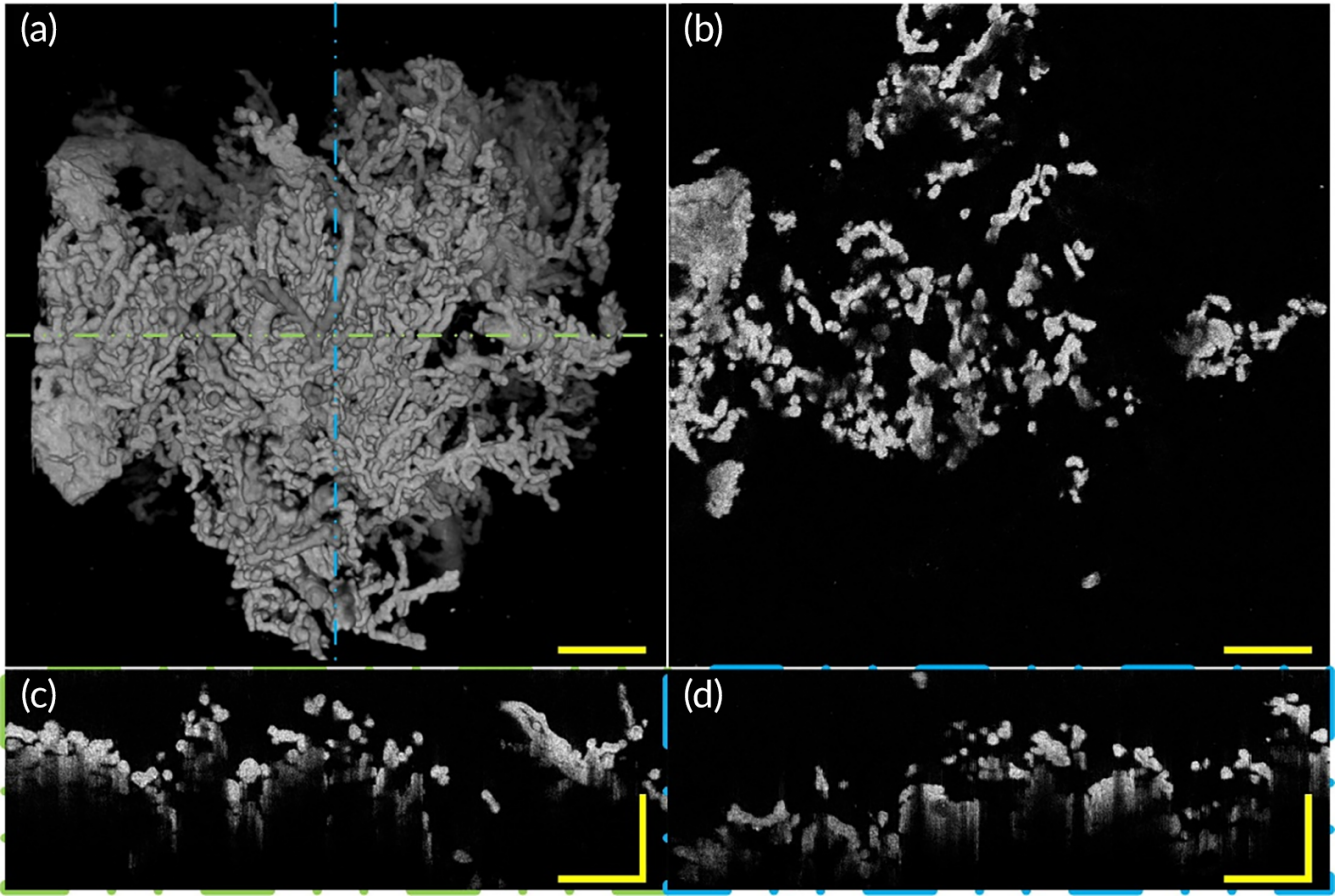
3D morphology and section images of placenta villi with GH via OCT. (a) Top view of 3D OCT morphology shows placental intermediate villi with GH are smaller in diameter and longer in length and the tree structure morphology characteristics of placenta with GH is different from the normal placenta, with more slender intermediate villi and fewer terminal villi which means poorly‐branched villous tree. (b) A representative OCT en‐face image of placenta villi with GH. (c) A representative rescaled B‐scan image of placental villi corresponding to the position indicated by the green line in (a). (d) A representative rescaled B‐scan image of placental villi corresponding to the position indicated by the blue dashed line in (a). All scale bars are 500 μm. GH, gestational hypertension; OCT, optical coherence tomography

Here we revealed a sample with mild GH diagnosed at 34 gestational weeks. Figure 6a–d and Video S2 reveal the morphology of a placenta with GH. A top view (Figure 6a) of a 3D OCT image of the placenta with GH revealed villous tree structure characteristics such as villi thickness, branches, and length. A representative OCT en‐face image of GH placental villi is shown in Figure 6b. In longitudinal‐plane B‐scan images (Figure 6c,d) corresponding to the positions represented by the green and blue dashed lines in Figure 6a, respectively, diameter change among villi of GH appeared to be smaller than healthy pregnancy. Combined with the 3D morphological image, we can distinguish that many villi in the B‐scan images are intermediate villi whose diameters are smaller than their counterparts in healthy pregnancy. We observed that the GH placenta has slender and small villi, with long and slender intermediate villi and fewer terminal villi. Villous tree with GH is poorly branched and fewer terminal villi sprout from intermediate villi which can be observed via Figure 6a. These characteristics extracted via the OCT imaging platform coincided with the aforementioned HDP placental villi morphological features. The morphological characteristics of the villi with GH we obtained are consistent with those found by other research methods, indicating that OCT three‐dimensional imaging can be used as a new method to supplement the research on the pathology of placental villi. In addition, more 3D morphological features that the 2D histological sections method could not reveal including villi 3D morphology and villous tree branching condition can be revealed via our OCT imaging system.

### 3.4 Fetal growth restriction

Fetal growth restriction (FGR)^23^ is defined as the failure of a fetus to achieve its genetic growth potential. FGR is an important cause of premature delivery and stillbirth, which is also associated with neonatal morbidity and mortality, impaired health in childhood,^24^ and increased rate of coronary heart disease and related disorders, stroke, hypertension, and type 2 diabetes in later life.^25^ Placental insufficiency is a major cause of fetal growth restriction, and one of the reasons is abnormalities of placental villous architecture.^26^, ^27^ Traditional pathological studies of FGR have suggested that both the weight and its diameter of the placenta are significantly smaller than in healthy pregnancy, with focal necrosis or fibrosis of the villi, and reduced number and diameter of intervillous capillarization, suggesting that primary villous dysplasia may be a potential contributor to fetal growth restriction.

**FIGURE 7:**
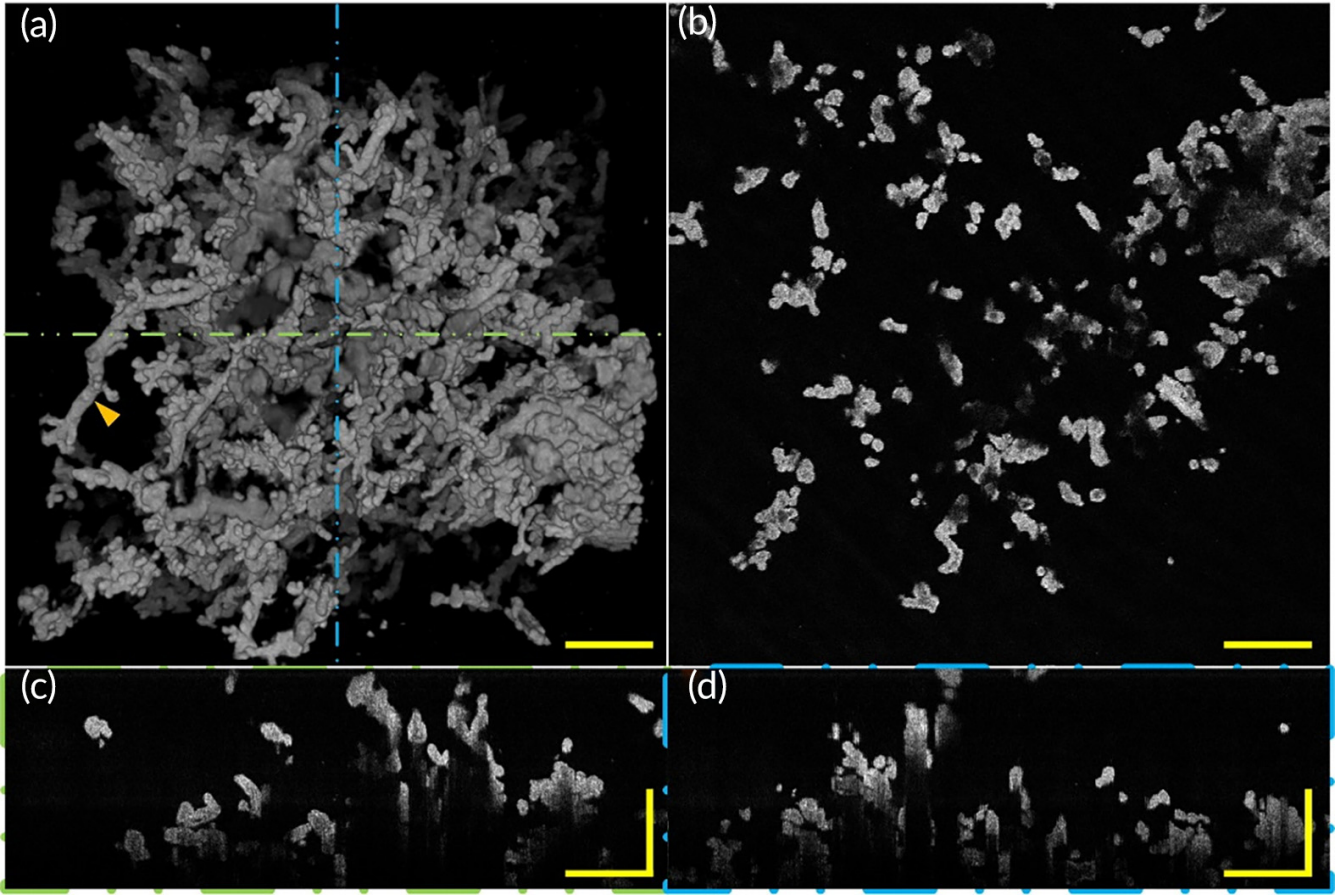
3D morphology and section images of placenta villi with FGR via OCT. (a) Top view of 3D OCT morphology shows placenta with FGR has relatively small volume terminal villi and the tree structure of the placenta with FGR is different than that of the normal placenta, with fewer villi branches and shorter intermediate villi. Representative intermediate villi with branched terminal villi (orange arrow). (b) A representative OCT en‐face image of placental villi with FGR. (c) A representative rescaled B‐scan image of placental villi corresponding to the position indicated by green dashed lines in (a). (d) A representative rescaled B‐scan section image of placental villi corresponding to the position indicated by blue dashed lines in (a). All scale bars are 500 μm. FGR, fetal growth restriction; OCT, optical coherence tomography

Here we employed our 3D imaging platform to reveal the villi morphology of placentae with FGR. Figure 7a–d and Video S3 show the morphology of a placenta with FGR. A top view (Figure 7a) of a 3D OCT image of the placenta with FGR showed villous tree structure morphology. A representative OCT en‐face section image of FGR placental villi is shown in Figure 7b. In longitudinal‐plane B‐scan images (Figure 7c,d) corresponding to the positions represented by the green and blue dashed lines in Figure 7a, respectively, the villi diameter of FGR appeared to be smaller than that of healthy placenta villus. As seen intuitively from the images, the placenta of FGR has smaller villi, with poorly‐branched intermediate villi and smaller in volume terminal villi. Representative poorly‐branched intermediate villi with fewer terminal villi are indicated by the orange arrows in Figure 7a. We can intuitively find that intermediate villi of FGR were smaller in diameter and villous trees were poorly branched. Compared with the normal placentae, the FGR placenta has slender intermediate villi, fewer terminal villi, and poorly branched villous trees. These morphology characteristics are also coincidental with the results of other methods. The results of our experiments show that villi morphological information which can be indicated by 2D means can be perfectly revealed via our OCT imaging system, and we can also further reveal some 3D morphological information of placental villi that cannot be extracted by 2D methods. Villous tree with FGR has poorly branches and chorionic membrane surface area are relatively smaller than that of healthy pregnancy, these maybe influence feto‐maternal material exchange and further lead to growth limitation of the fetus.

### 3.5 Gestational diabetes mellitus

Gestational diabetes mellitus (GDM), one of the most common disorders of pregnancy, occurs generally by the end of the second and beginning of the third trimester of pregnancy. And it is characterized by abnormal glucose metabolism affecting the mother and fetus. The maternal blood of pregnant women with gestational diabetes mellitus is hyperglycemia,^28^ it is believed that maternal hyperglycemic status can affect placental villus morphology and capillary distribution, by altering the diameter of placental villi and their capillaries, causing placental vascular disorders, which in turn affect fetal development and growth.^29^ Histological sections and SEM studies of placental villi with GDM have found that the terminal villi show some structural lesions, including a significantly lower number of villi with shorter, malaligned villi. Typical gestational diabetes has poor terminal villus maturation, and increased immature villi. Among them, immature villi have a bulb‐like shape and poorly developed capillaries with a distinct reticular interstitium and fluid‐filled interstitial channels, usually about 100–200 μm in diameter and sometimes up to 400 μm.

**FIGURE 8:**
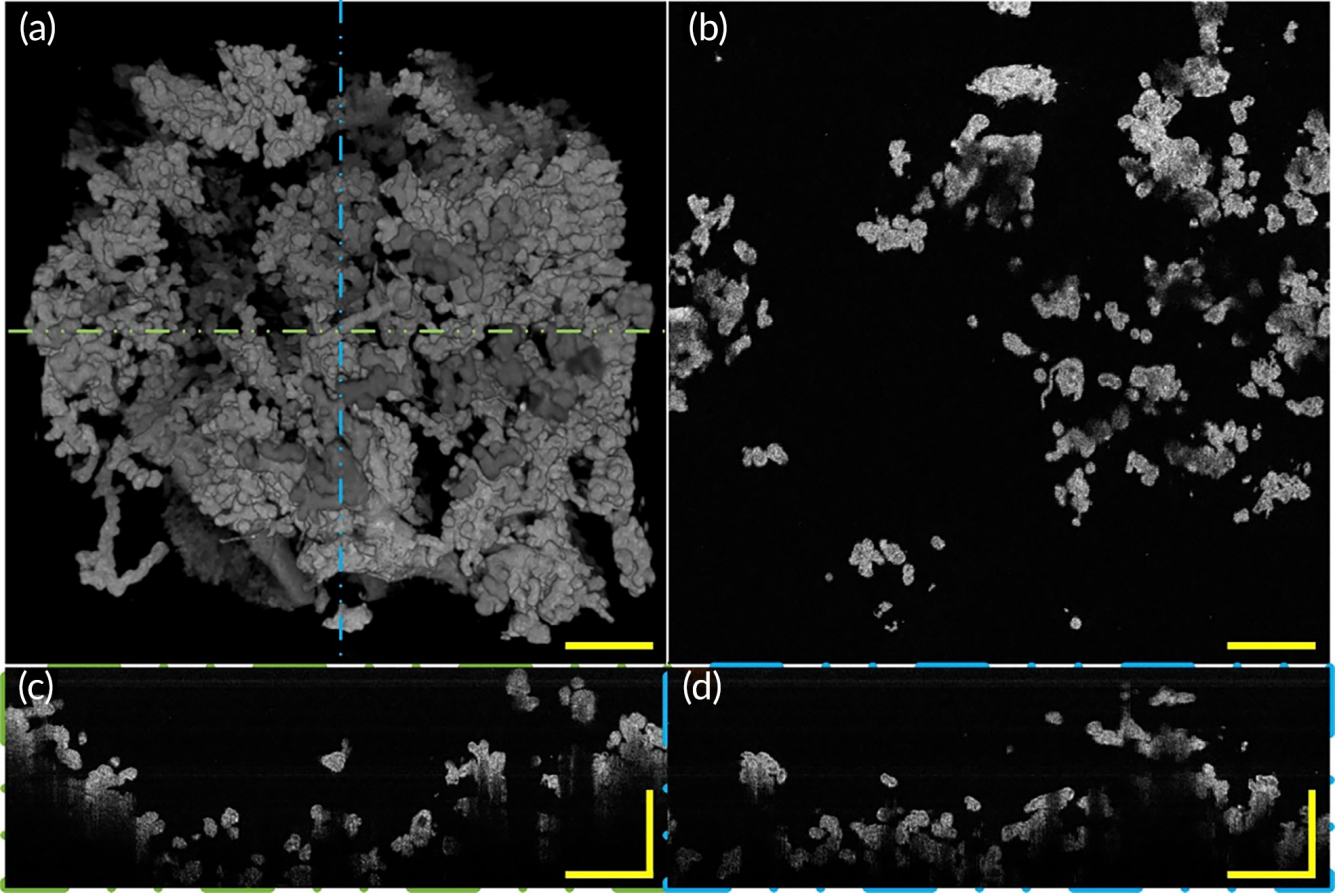
3D morphology and section images of placenta villi with GDM via OCT. (a) Top view of 3D OCT morphology shows villous trees of placenta with GDM were well‐branched. (b) A representative OCT en‐face section image of placental villi with GDM. (c) A representative rescaled B‐scan section image of placental villi corresponding to the position indicated by the green line in (a). (d) A representative rescaled B‐scan section image of placental villi corresponding to the position indicated by the blue dashed line in (a). All scale bars are 500 μm. GDM, gestational diabetes mellitus; OCT, optical coherence tomography

Figure 8a–d and Video S4 show the morphology of a placenta with GDM. A representative OCT en‐face image of GDM placental villi is shown in Figure 8b. In longitudinal‐plane B‐scan images (Figure 8c,d) corresponding to the positions represented by the green and blue dashed lines in Figure 8a, respectively, revealed villous tree structure 3D morphology of placenta with GDM. In Figure 8c corresponding to the position represented by the green line in Figure 8a, we can find that the terminal villi diameter of GDM appeared to be larger than that of healthy pregnancy. We observed that the GDM placenta had larger and shorter villi, and the villi diameter was relatively larger than that of healthy pregnancies, with densely terminal villi. Villous trees of GDM were well‐branched with each intermediate villi with many‐branched terminal villi. Undoubtedly, our 3D placental villi images have greater advantages than traditional 2D pathological research methods in revealing the morphology of placental villi. Our results can be consistent with previous research methods, and we can reveal villi 3D morphological features from a more clear and intuitive perspective. These well‐branched villous tree structures and relatively large villi diameter indicate that the placenta with GDM was overgrown to some extent. These morphology changes of the placenta with GDM may be one of the reasons that cause adverse obstetric outcomes including preterm labor, cesarean‐section, and macrosomia.

### 3.6 Multiple comorbidities of FGR and GDM

**FIGURE 9:**
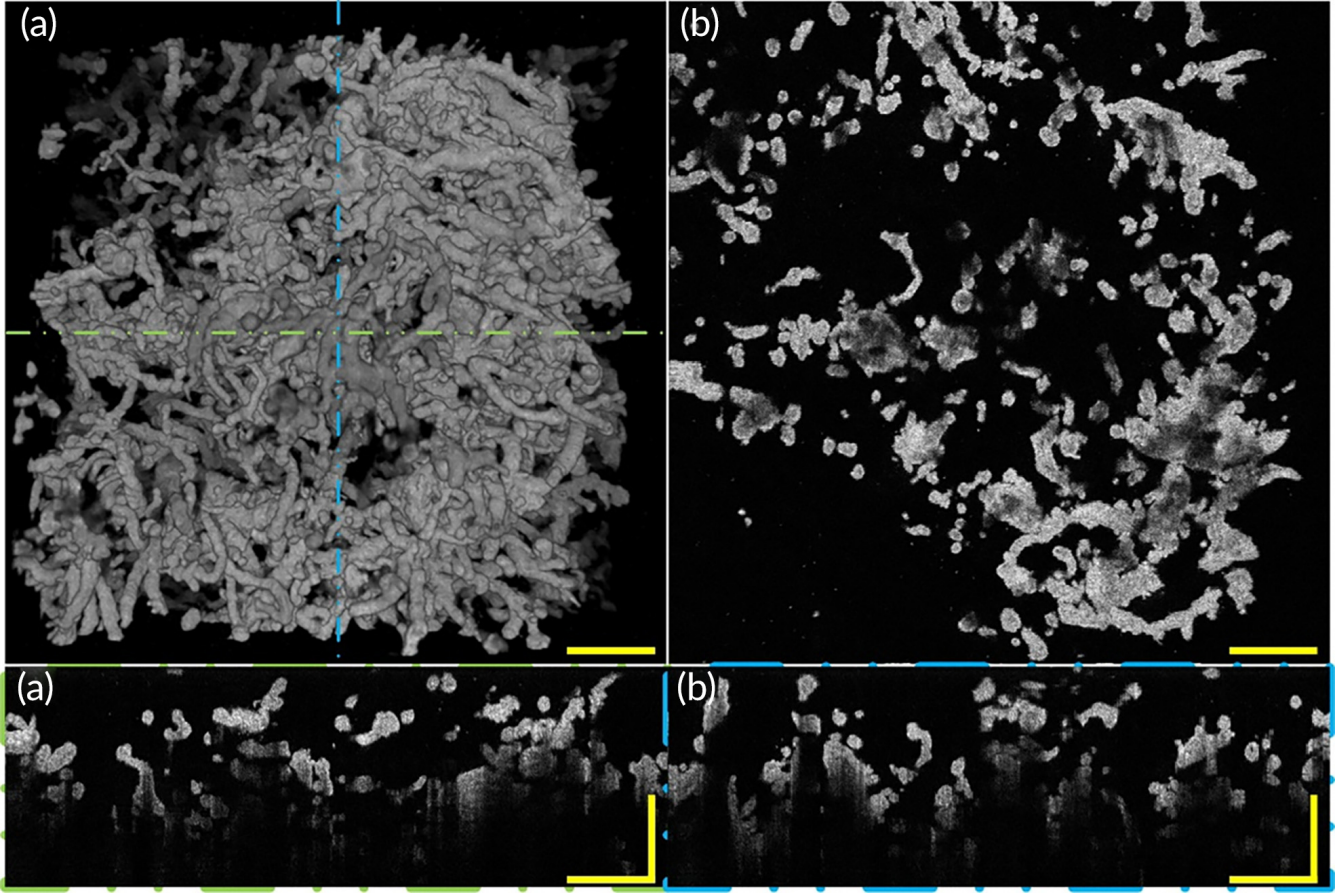
3D morphology and section images of placenta villi with GDM and FGR via OCT. (a) Top view of 3D OCT morphology of placenta villi with GDM and FGR. (b) A representative OCT en face image of placenta villi with GDM and FGR. (c) A representative rescaled B‐scan image of placental villi corresponding to the position indicated by the green line in (a). (d) A representative rescaled B‐scan image of placental villi corresponding to the position indicated by the blue dashed line in (a). All scale bars are 500 μm. FGR, fetal growth restriction; GDM, gestational diabetes mellitus; OCT, optical coherence tomography

During pregnancy, two or more complications occurred simultaneously make the pregnancy more complex to diagnose and treat. Therefore, it is of practical significance to study the placenta morphology that suffers from multiple pregnancy complications at the same time. Here 3D morphology of a placenta with FGR and GDM was revealed via the OCT imaging platform. Figure 9a–d and Video S5 show the morphology of a placenta with FGR and GDM. A top view (Figure 9a) of a 3D OCT image revealed villous tree structure 3D morphology of placenta with FGR and GDM. In a longitudinal‐plane B‐scan image (Figure 9c) corresponding to the position represented by the green line in Figure 9a, we can observe that the terminal villi diameter of GDM with FGR is smaller. A representative OCT en‐face image of FGR with GDM placental villi is shown in Figure 9b. In B‐scan images (Figure 9c,d) corresponding to the positions represented by the green and blue dashed lines in Figure 9a, respectively, we observed placental villus trees with FGR and GDM were poorly branched, each intermediate villi has few branched terminal villi which coincidence with FGR villus tree morphological characteristics. These morphological characteristics of placenta with FGR and GDM such as poorly branched villus tree, smaller in diameter intermediate villi, and maldeveloped terminal villi are all met FGR placenta villi morphological characteristics indicate this placenta villus sample is mainly influenced by FGR. This placenta sample complicated by FGR and a mild GDM supports our results which indicate this placenta villi morphology is mainly subject to FGR.

### 3.7 Quantitative analysis of placental villi

**FIGURE 10:**
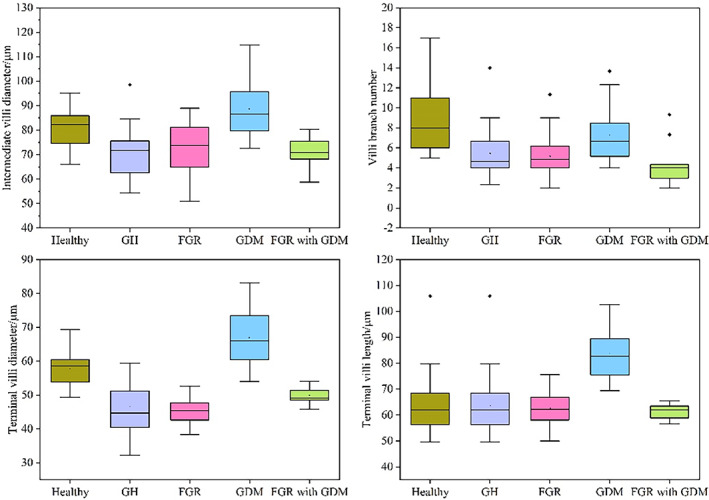
Boxplots of villi morphological parameters of the healthy and complicated placenta. (a) Comparison of intermediate villi diameter between healthy and complicated placenta. (b) Comparison of villi branch number between healthy and complicated placenta. (c) Comparison of terminal villi diameter between healthy and complicated placenta. (d) Comparison of terminal villi length between healthy and complicated placenta

Here we quantitatively evaluated the 3D tree structure morphological parameters of placenta villi, including intermediate villi diameter (IVD), terminal villi diameter (TVD), terminal villi length (TVL), and villi branch number (VBM), as Figure 10 shows. Figure 10a–d showed the comparisons of intermediate villi diameters between healthy and complicated placenta, the villi branch number between healthy and complicated placenta, and the terminal villi diameter between healthy and complicated placenta, and the terminal villi length between healthy and complicated placenta.

Here we used placental villi quantification results of healthy and complicated placental samples when acquiring the boxplots. For healthy placenta and placenta complicated with GH, GDM, and FGR, we used four placental samples from different pregnancies for each condition, and for each placental sample, we quantified six representative regions to acquire universal and reliable quantification results. We used two placenta samples of multiple comorbidities of FGR and GDM to acquire quantification results. For these multiple comorbidities placenta, we performed more densely quantification sampling and selected more quantification regions to acquire relatively accurate results with fewer placenta samples.

Boxplots of quantitative analysis results (Figure 10) show that morphological characteristics of placenta villi were consistent with what we observed from 3D images and cross‐section images of OCT. Intermediate villi diameter of the healthy placenta are larger than placenta with GH, FGR, and FGR with GDM, and lower than placenta with GDM, as Figure 10a shows. Villi branch number results show that villi of healthy placenta have the most branch number each villus. Placenta samples complicated with GDM have more branch numbers than placenta samples with GH, FGR, and FGR with GDM, as Figure 10b shows. Compared with healthy placental samples and other complicated placental samples in our study, the placenta complicated with GDM has the largest terminal villi diameter, as Figure 10c shows. Healthy placenta terminal villi have a larger diameter than complicated placental terminal villi except for placenta with GDM. Placenta samples with GDM have longer terminal villi than healthy and other complicated placenta samples we studied, as Figure 10d shows. These measured data agree with placental villi morphological characters extracted from 3D OCT images and cross‐section images before.

The results of placental villi morphological parameters quantification are shown in Table 1. Compared with healthy placenta, mean IVD of complicated placenta show statistically significant differences. In detail, mean IVD of placenta complicated with GH (*p* < 0.001), FGR (*p* = 0.02) and multiple comorbidities of FGR and GDM (*p* < 0.001) are smaller than healthy placenta, while larger when placenta complicated with GDM (*p* = 0.01). Mean TVL of placenta complicated with GDM (*p* < 0.001) are significantly larger than healthy placenta, while placenta complicated with GH (*p* < 0.001), FGR (*p* = 0.01), multiple comorbidities of FGR and GDM (*p* < 0.001) are significantly smaller than healthy placenta. Mean TVD of placenta complicated with GDM (*p* < 0.01) is significantly larger than healthy placenta, while all other complicated placenta is significantly smaller than the healthy placenta. Mean VBM of placenta complicated with GH (*p* < 0.01), FGR (*p* < 0.01), and multiple comorbidities of FGR and GDM (*p* < 0.01) are significantly smaller than the healthy placenta. Mean VBM of placenta complicated with GDM (*p* = 0.068) is decreased compared to healthy placenta without statistical significance. All parameters compared to healthy placenta without statistical significance are marked in yellow.

**TABLE 1:** Measurement results and comparison of placental villi parameters in various diseases

Placenta type	Mean IVD ± SD (μm)	Mean TVL ± SD (μm)	Mean TVD ± SD (μm)	Mean VBM ± SD (μm)
	*p*‐value	*p*‐value	*p*‐value	*p*‐value
Healthy	81.5 ± 7.6	69.0 ± 6.0	57.6 ± 5.2	8.8 ± 3.3
GDM	88.7 ± 10.8	83.6 ± 9.3	66.9 ± 8.6	7.2 ± 2.6
	*p* = 0.01	*p* < 0.001	*p* < 0.001	*p* = 0.068
GH	71.4 ± 10.3	56.6 ± 4.1	46.5 ± 7.2	5.4 ± 2.4
	*p* < 0.001	*p* < 0.001	*p* < 0.001	*p* < 0.001
FGR	72.6 ± 10.5	62.6 ± 6.0	45.5 ± 3.8	5.5 ± 2.1
	*p* = 0.02	*p* = 0.01	*p* < 0.001	*p* < 0.001
FGR with GDM	70.9 ± 5.7	61.4 ± 2.9	49.8 ± 2.6	4.4 ± 2.0
	*p* < 0.001	*p* < 0.001	*p* < 0.001	*p* < 0.001

Abbreviations: FGR, fetal growth restriction; GDM, gestational diabetes mellitus; GH, gestational hypertension; IVD, intermediate villi diameter; TVD, terminal villi diameter; TVL, terminal villi length; VBM, villi branch number.

## 4 DISCUSSION AND CONCLUSION

In this study, we established a 3D imaging system via OCT to acquire 3D label‐free images of villous tree structures of the placenta with three different gestational complications and healthy pregnancies. We revealed and extracted the characteristics of placental villous tree structures of three different complications and healthy pregnancies: villi of the healthy placenta are well developed and densely branched; compared with healthy placenta villi, placenta villi with GH have smaller diameter intermediate villi with minimal branching and accompanied by a lack of terminal villi and smaller terminal villus diameter; FGR placental villous tree are poorly‐branched with relatively smaller in diameter villi; GDM placenta villi has larger in diameter villi and relatively well‐branched villous tree which was better than other complicated placenta but worse than the healthy placenta. These results are consistent with pathological studies. In addition, 3D morphology of placental villus with multiple comorbidities has been revealed and found that placenta with FGR and a mild GDM simultaneously has morphological characteristics such as smaller in diameter terminal villi and poorly‐branched intermediate villi of FGR. These morphological characteristics supported by quantitative analysis indicate placenta complicated with FGR and GDM are more similar in morphology to placenta complicated with FGR, showing the morphology revealing the power of OCT. Furthermore, 3D OCT images provide a panoramic view of multi‐diseased placenta villi and reveal morphology characteristics that are not available in 2D imaging methods.

Quantitative assessment of the 3D tree structure of placental villi, including the diameters of intermediate and terminal villi, length of intermediate villi, and the number of villi branches is of great importance because placenta morphology and development are highly relevant to these parameters. Currently, imaging platforms using histological sections, SEM, confocal laser scanning microscopy (CLSM), synchrotron X‐ray imaging, and Micro‐CT have been used for imaging and analysis of morphological parameters including villi diameter of placental villi. However, they are unable to resolve the entire, large field of view placenta villous tree structure and unable to quantify the number of villus tree branches. In this study, we can quantify placenta villi diameter more conveniently and accurately compared with the histological sections method. Moreover, our 3D OCT imaging data makes it possible to quantify villus length and the number of villus tree branches in a way that was technically difficult with histological section methods. In addition, quantitative analyses of placental villi of healthy and complicated pregnancies showed that 3D OCT imaging data has great advantages in quantifying villi parameters such as villi diameter, length, and branch numbers. Villi diameter quantification based on 3D OCT data yielded more accurate results more conveniently. Furthermore, 3D OCT images provide a unique view of the placenta villus tree and offer a 3D placental villus tree structure which is not available in 2D imaging methods, making it possible to quantify the number of villus branches easily, and accurately and quickly. Quantitative analysis results of healthy and complicated placental villi samples are consistent with what we have acquired from 3D OCT images and cross‐section images.

The current quantitative assessment of placenta villi is conducted manually and quantitative speed and accuracy would be affected by human factors. Quantitative analysis's speed and accuracy can be further improved by combining with artificial intelligence methods. In the future, the method based on artificial intelligence will greatly improve quantitative analyses efficiency and achieve higher precision, which we are focusing on now. At present, the total amount of placental samples in our study is relatively insufficient, and further research will obtain more reliable and accurate results. Here the pixel‐resolution rescale operations were performed on the 3D image volumes, and a specific index mentioned in Section 2.3 was chosen to numerically rescale the axial pixel resolution to achieve that the acquired 3D image volumes have the same pixel resolution along different axis directions, so it can be more convenient to observe and measure the 3D morphology of human placental villi. Meanwhile, here we assumed the refractive index of the sample over a wide range of wavelengths as ~1.38, following the References 30, 31 to estimate the OCT axial optical resolution in the sample, which was ~1.7 μm.

In summary, we have revealed and extracted 3D morphological characteristics of multi‐diseased and healthy human placenta via our customized OCT imaging system and demonstrated possible influence mechanism of placental villous tree morphology with different gestational complications on feto‐maternal material exchange. In addition to villous tree structure visualization, our study provides complementary 3D information to better characterize villous tree structure morphology as a whole, yielding an intuitive and non‐invasive morphological revealing of human placenta villi via OCT. Moreover, our customized 3D imaging system via OCT could be reliably utilized to reveal inner villi structures such as capillary and membrane in villi by using a higher magnification microscope, suggesting a promising study prospect of placental villous capillary morphology. Overall, OCT not only reinforced the depth‐resolved distinct morphologies noted by histological sections but also uncovered key 3D morphological features which conventional 2D methods cannot provide such as villi morphology and villous tree branching conditions, and that further revealed morphology differences from each of the three gestational complications and healthy pregnancy. Our study proves that 3D morphological revealing of multi‐diseased human placenta villous tree structure via OCT can provide morphological characteristics of villous tree such as villi morphology and branching conditions which conventional 2D methods cannot provide. This morphological information on different gestational complications and healthy pregnancies could be of reference significance for future research on the relationship between the placenta and gestational complications and can provide a brand‐new perspective to help study the relationship between pregnancy diseases and feto‐maternal material exchange.

## AUTHOR CONTRIBUTIONS

**Guangming Ni:** Conceptualization (equal); data curation (equal); formal analysis (equal); funding acquisition (equal); investigation (equal); methodology (lead); project administration (equal); resources (equal); writing – original draft (equal); writing – review and editing (equal). **Junming Zhong:** Data curation (equal); methodology (supporting); software (equal); visualization (equal); writing – original draft (equal). **Xuemei Gao:** Data curation (equal); formal analysis (supporting); resources (supporting); software (supporting); writing – original draft (supporting). **Wenjing Wang:** Formal analysis (supporting); resources (supporting); software (supporting). **Renxiong Wu:** Data curation (supporting); software (supporting); writing – original draft (supporting). **Xiaoshan Wang:** Data curation (supporting); funding acquisition (supporting). **Yao Xie:** Conceptualization (equal); data curation (supporting); formal analysis (equal); methodology (equal); resources (equal); writing – review and editing (supporting). **Yong Liu:** Conceptualization (equal); formal analysis (equal); investigation (equal); project administration (equal); resources (equal); supervision (equal); writing – review and editing (equal). **Jie Mei:** Conceptualization (equal); investigation (equal); methodology (equal); project administration (equal); resources (equal); supervision (equal); writing – review and editing (equal).

## FUNDING INFORMATION

National Natural Science Foundation of China (61905036); China Postdoctoral Science Foundation (2021T140090, 2019M663465); Fundamental Research Funds for the Central Universities (University of Electronic Science and Technology of China) (ZYGX2021J012, ZYGX2021YGCX019); Chengdu Science and Technology Innovation research and development project (2021‐YF05‐02143‐SN); Clinical Research and Transformation Fund of Sichuan Provincial People's Hospital (2021LY25).

## CONFLICT OF INTEREST

The authors declare no conflicts of interest.

5

### PEER REVIEW

The peer review history for this article is available at https://publons.com/publon/10.1002/btm2.10372.

## Supporting information


**Video S1** Supporting information

**Video S2** Supporting information

**Video S3** Supporting information

**Video S4** Supporting information

**Video S5** Supporting information

## Data Availability

Data underlying the results presented in this paper are not publicly available at this time but may be obtained from the authors upon reasonable request.
